# Evaluation of Artificial Intelligence–Based Grading of Diabetic Retinopathy in Primary Care

**DOI:** 10.1001/jamanetworkopen.2018.2665

**Published:** 2018-09-28

**Authors:** Yogesan Kanagasingam, Di Xiao, Janardhan Vignarajan, Amita Preetham, Mei-Ling Tay-Kearney, Ateev Mehrotra

**Affiliations:** 1 Australian e-Health Research Centre, Commonwealth Scientific and Industrial Research Organisation, Perth, Western Australia, Australia; 2 Department of Health Care Policy, Harvard Medical School, Boston, Massachusetts; 3 GP Super Clinic at Midland Railway Workshops, Midland, Western Australia, Australia; 4 Department of Ophthalmology, Royal Perth Hospital, Perth, Western Australia, Australia

## Abstract

**Importance:**

There has been wide interest in using artificial intelligence (AI)–based grading of retinal images to identify diabetic retinopathy, but such a system has never been deployed and evaluated in clinical practice.

**Objective:**

To describe the performance of an AI system for diabetic retinopathy deployed in a primary care practice.

**Design, Setting, and Participants:**

Diagnostic study of patients with diabetes seen at a primary care practice with 4 physicians in Western Australia between December 1, 2016, and May 31, 2017. A total of 193 patients consented for the study and had retinal photographs taken of their eyes. Three hundred eighty-six images were evaluated by both the AI-based system and an ophthalmologist.

**Main Outcomes and Measures:**

Sensitivity and specificity of the AI system compared with the gold standard of ophthalmologist evaluation.

**Results:**

Of the 193 patients (93 [48%] female; mean [SD] age, 55 [17] years [range, 18-87 years]), the AI system judged 17 as having diabetic retinopathy of sufficient severity to require referral. The system correctly identified 2 patients with true disease and misclassified 15 as having disease (false-positives). The resulting specificity was 92% (95% CI, 87%-96%), and the positive predictive value was 12% (95% CI, 8%-18%). Many false-positives were driven by inadequate image quality (eg, dirty lens) and sheen reflections.

**Conclusions and Relevance:**

The results demonstrate both the potential and the challenges of using AI systems to identify diabetic retinopathy in clinical practice. Key challenges include the low incidence rate of disease and the related high false-positive rate as well as poor image quality. Further evaluations of AI systems in primary care are needed.

## Key Points

**Question:**

How will an artificial intelligence (AI)–based grading system for diabetic retinopathy perform in a real-world clinical setting?

**Findings:**

In a diagnostic study evaluating 193 patients (386 images), the AI system judged 17 as having diabetic retinopathy of sufficient severity to require referral. While the system correctly identified the 2 patients with true disease (severe diabetic retinopathy), the positive predictive value was only 12%, with 15 patients misclassified as needing referral.

**Meaning:**

Grading of diabetic retinopathy using AI has both potential benefits and challenges, and further study in real-world settings is needed.

## Teaser

This diagnostic study evaluates an artificial intelligence (AI)–based system for diagnosing diabetic retinopathy using retinal images in a primary care setting vs the gold standard of diagnosis by an ophthalmologist.

## Introduction

Diabetic retinopathy (DR), if untreated, leads to progressive visual impairment and eventual blindness.^1^ Timely identification and referral to ophthalmologists could reduce blindness and disease complications. Those with poorly controlled diabetes should be screened for DR at least annually^2^; however, only half of such patients receive screening.^3^ Screening currently requires referral to an eye specialist, and patients may not visit the specialist because of logistical barriers, cost of the visit, or lack of an eye specialist in their community.

One method of improving access to DR screening is for primary care practices to obtain color fundus images and send these to ophthalmologists or optometrists for reading.^4^ While such programs increase screening rates,^5^ there are logistical barriers, costs, and time delays in having the images read by ophthalmologists or optometrists.

These limitations have driven interest in computer assessment of images through fully automated artificial intelligence (AI)–based grading systems. Such a system would decide in real time whether a patient needs referral and could potentially be much cheaper than having eye experts conduct screening. Several studies have used repositories of retinal images to test the performance of AI grading systems in detecting DR,^6,7,8,9,10^ and in April 2018 the US Food and Drug Administration approved an AI algorithm, developed by IDx, used with Topcon Fundus camera (Topcon Medical) for DR identification.^11^

Despite enthusiasm about the potential of AI-based grading systems, to our knowledge, there has never been an evaluation of the performance of an AI system in a real-world clinical setting. In this pilot study, we describe the performance of an AI system in a primary care practice.

## Methods

The study design and patient information and informed consent forms for study participants were approved by the Human Research Ethics Committee at the University of Notre Dame, Fremantle, Australia, and patients provided written informed consent. We conducted the trial according to the Standards for Reporting of Diagnostic Accuracy (STARD) reporting guideline.

### AI-Based Grading System for DR

Our AI system is based on deep learning and rule-based models for DR. It was developed and evaluated based on manually outlined pathologies using color fundus images from several training data sets (altogether 30 000 images) including DiaRetDB1^12^ and Kaggle^13^ (EyePACS) databases and our own Australian Tele-eye care DR database. The model was retrained using the images from the 3 data sets. The deep learning model adopted a deep convolutional neural network model. We used the convolutional neural layers from the deep learning model Inception-v3 as our base model and connected it to our customized top model with several fully connected layers for the purpose of DR image classification. By applying transfer-learning technology, the model training process includes the following steps: (1) manually classify the selected image data into 2 categories, DR disease and no DR disease; (2) divide the categorized image data into 2 parts, training data set (80% of the total) and test data set (20%) and keep the balance of the 2 categories in each data set; (3) normalize all the images and resize them to the dimension of 299 × 299 pixels; (4) load the pretrained base model weights and use the training data set to train the top model initially; (5) use the training data set to retrain the whole model; and (6) monitor the accuracy and loss on the training data set and test data set and achieve the best model. The rule-based model adopted selection criteria results in 3 outcomes: (1) a binary identification of disease or no disease for clinically significant DR, (2) identification of specific pathologies (eg, microaneurysms and exudates) related to DR, and (3) the severity of DR based on the International Clinical Diabetic Retinopathy Disease Severity Scale criteria.^14^ The AI system is compatible with most retinal imaging cameras (eg, Canon, Zeiss, and DRS cameras).

The image quality control system used deep learning techniques to check the quality of the images. We manually classified selected images from the data sets into 2 classes: adequate image quality for DR grading and inadequate image quality for DR grading. Then we used the only adequate quality images to train the convolutional neural network model. However, there were some images whose quality was ambiguous between adequate and inadequate, which was expected to influence some outcomes.

### Deployment in a Primary Care Practice

We deployed the AI system for 6 months (December 1, 2016, to May 31, 2017) at a primary care practice in Midland, Western Australia, that employed 4 primary care physicians. The tele-retinal and AI system includes a color fundus camera (Canon CR-2 AF), a cloud computing server, and a web application server.

Over roughly 1 to 2 weeks we trained 2 nurses to use the fundus camera and our tele-retinal screening software. All patients with diabetes seen at the primary care clinic were invited to participate in the study. Macula-centered images were acquired and 1 to 3 images per eye were allowed depending on the image quality (confirmed by quality control software). After completing the imaging process, the system sent the patient information and related images to a web server using Digital Imaging and Communications in Medicine format. The DR grading system provided a binary disease or no-disease DR grade to the primary care physician via an email. Patients with moderate or severe DR were referred to an ophthalmologist immediately.

All images were also sent to an ophthalmologist for evaluation using our tele-retinal system. If the ophthalmologist’s reading differed from the AI system’s, the ophthalmologist’s reading was relayed to the physician.

### Statistical Analysis

The binary reading (disease or no disease) by an ophthalmologist was used as the gold standard and compared with the grading obtained from our AI system. The sensitivity of the disease grading was true-positive/(true-positive + false-negative), specificity was true-negative/(true-negative + false-positive), positive predictive value was true-positive/(true-positive + false-positive), and negative predictive value was true-negative/(true-negative + false-negative).

## Results

During the study period, the practice saw 216 patients with diabetes. Of the 193 patients who agreed to DR screening, 93 (48%) were women. The mean (SD) age was 55 (17) years with a range of 18 to 87 years.

The nurse took approximately 10 to 15 minutes to obtain images for both eyes, and the AI system provided reading outcomes in less than 3 minutes. Three hundred eighty-six images were reviewed.

Based on grading by an ophthalmologist, of the 193 patients, 183 had no signs of retinopathy, 8 had mild nonproliferative DR, and 2 had clinically significant DR (1 with moderate nonproliferative DR, 1 with severe nonproliferative DR). The 2 patients with moderate or severe disease required referral to an ophthalmologist (Table 1).

**Table 1.** Grading Outcome From the Ophthalmologist and Artificial Intelligence System

Source	No.		
	Positive Diagnosis	Negative Diagnosis	False-Positives
Artificial intelligence	17	176	15
Ophthalmologist	2	191	0

Our AI system classified 17 patients as having clinically significant DR and 176 without disease. The system classified the 2 patients with true moderate and severe DR as having disease, indicating that they should be referred to ophthalmologists. It also identified all 8 mild DR cases correctly. Of the 17 patients classified as having clinically significant disease, 15 were false-positives. This resulted in a specificity of 92% (95% CI, 87%-96%) and a positive predictive value of 12% (95% CI, 8%-18%) (Table 2).

**Table 2.** Sensitivity, Specificity, and Positive Predictive Values

Characteristic	Value (95% CI), %
Sensitivity	2 patients with severe disease were correctly identified^a^
Specificity	92 (87-96)
Negative predictive value	100^a^
Positive predictive value	12 (8-18)

^a^ 95% CI cannot be generated.

There were several factors that led to the 15 false-positive results. Six had drusen that were similar in appearance to exudates. Other false positives were driven by dirty lens reflections or uneven light exposure at the rim of images that our image quality control process could not fully identify. The AI system also identified exudates that were sheen reflections around the optic disc, the papillomacular area, and the macula.

## Discussion

We evaluated the performance of an AI system that reads retinal images to identify DR in a real-world clinical setting. The system was successfully deployed and detected 2 patients with severe DR requiring referral. Though there was a limited sample size, the AI system was effective in ruling out disease. However, the system had a high rate of false-positives with a specificity of 92% and positive predictive value of just 12%.

The specificity of the deployed system (92%) is similar to our prior validation using a database of retinopathy images (93%) and similar to other AI systems for reading retinopathy images (93.4%).^9,10^ The high rate of false-positives was driven by the low incidence of disease (2 of 193 [1%]). Prior validations of AI systems for identifying DR have used data from retinal image databases, and images were preselected such that the incidence of disease was much higher (roughly 1 of 3). On average, when the disease incidence is lower, the positive predictive value will also be lower. This is consistent with other screening programs where false-positives are common, such as mammograms.^15^ The low incidence rate of DR we observed in our study is the norm in primary care; therefore, false-positives are likely to be an issue unless the specificity of our system or other systems is much higher. Given this limitation, we believe retinopathy images identified as having illness by an AI system should be reviewed by an ophthalmologist before a referral is made.

Despite these limitations, we believe the AI system has potential for improving the efficiency of screening for DR in primary care. Roughly 92% of all patients were immediately told at their primary care practice they had no DR and therefore no referral was needed. In this case, the number of patients that would have to be reviewed by an ophthalmologist was less than 10%. The ability to provide real-time eye screening at familiar primary care physician practices has many practical advantages, including comprehensive chronic disease management at a single location for patients with diabetes. There is also the potential for the AI system to be improved. Further training of the AI system to differentiate drusen, sheen reflections, and exudates can improve the specificity.

### Limitations

There were 2 key limitations of this study. The first is the small sample size and that only 2 of the screened patients had clinically significant disease. The second is generalizability. Our study was limited to 1 primary care practice in Western Australia and used a single AI system.

## Conclusions

Our evaluation demonstrates both the promise and challenges of using AI systems to identify DR in clinical practice. Evaluations of AI systems should be conducted in real-world clinical practice before they are deployed widely.

## References

[1] Zhang X, Saaddine JB, Chou C-F, Prevalence of diabetic retinopathy in the United States, 2005-2008. JAMA. 2010;304(6):-. doi:10.1001/jama.2010.1111 20699456 PMC2945293

[2] Kroenke K Telemedicine screening for eye disease. JAMA. 2015;313(16):1666-1667. doi:10.1001/jama.2015.107 25919530

[3] Murchison AP, Hark L, Pizzi LT, Non-adherence to eye care in people with diabetes. BMJ Open Diabetes Res Care. 2017;5(1):e000333. doi:10.1136/bmjdrc-2016-000333 28878930 PMC5574424

[4] Rosenberg JB, Tsui I Screening for diabetic retinopathy. N Engl J Med. 2017;376(16):1587-1588. doi:10.1056/NEJMe1701820 28423293

[5] Taylor CR, Merin LM, Salunga AM, Improving diabetic retinopathy screening ratios using telemedicine-based digital retinal imaging technology: the Vine Hill study. Diabetes Care. 2007;30(3):574-578. doi:10.2337/dc06-1509 17327323

[6] Abràmoff MD, Reinhardt JM, Russell SR, Automated early detection of diabetic retinopathy. Ophthalmology. 2010;117(6):1147-1154. doi:10.1016/j.ophtha.2010.03.046 20399502 PMC2881172

[7] Faust O, Acharya U R, Ng EYK, Ng K-H, Suri JS Algorithms for the automated detection of diabetic retinopathy using digital fundus images: a review. J Med Syst. 2012;36(1):145-157. doi:10.1007/s10916-010-9454-7 20703740

[8] Tozer K, Woodward MA, Newman-Casey PA Telemedicine and diabetic retinopathy: review of published screening programs. J Endocrinol Diabetes. 2015;2(4):1-10. doi:10.15226/2374-6890/2/4/00131 27430019 PMC4942133

[9] Gulshan V, Peng L, Coram M, Development and validation of a deep learning algorithm for detection of diabetic retinopathy in retinal fundus photographs. JAMA. 2016;316(22):2402-2410. doi:10.1001/jama.2016.17216 27898976

[10] Saha SK, Fernando B, Xiao D, Tay-Kearney M-L, Kanagasingam Y Deep learning for automatic detection and classification of microaneurysms, hard and soft exudates, and hemorrhages for diabetic retinopathy diagnosis. Invest Ophthalmol Vis Sci. 2016;57(12):5962.

[11] US Food and Drug Administration FDA permits marketing of artificial intelligence-based device to detect certain diabetes-related eye problems. https://www.fda.gov/NewsEvents/Newsroom/PressAnnouncements/ucm604357.htm. Published April 11, 2018. Accessed August 12, 2018.

[12] Lappeenranta University of Technology Diabetic Retinopathy Database and Evaluation Protocol http://www2.it.lut.fi/project/imageret/diaretdb1_v2_1/. Updated May 6, 2009. Accessed May 29, 2018.

[13] Kaggle Diabetic Retinopathy Database https://www.kaggle.com/c/diabetic-retinopathy-detection. Published July 27, 2015. Accessed May 29, 2018.

[14] Wilkinson CP, Ferris FL III, Klein RE, ; Global Diabetic Retinopathy Project Group Proposed international clinical diabetic retinopathy and diabetic macular edema disease severity scales. Ophthalmology. 2003;110(9):1677-1682. doi:10.1016/S0161-6420(03)00475-5 13129861

[15] Hofvind S, Geller BM, Skelly J, Vacek PM Sensitivity and specificity of mammographic screening as practised in Vermont and Norway. Br J Radiol. 2012;85(1020):e1226-e1232. doi:10.1259/bjr/15168178 22993383 PMC3611728

